# Pharmacy professionals’ experiences and perceptions of providing NHS patient medicines helpline services: a qualitative study

**DOI:** 10.1186/s12913-020-05182-w

**Published:** 2020-04-29

**Authors:** Matt Williams, Abbie Jordan, Jenny Scott, Matthew D. Jones

**Affiliations:** 1 Department of Pharmacy & Pharmacology, University of Bath, 5 West, Claverton Down, Bath, BA2 7AY UK; 2 Department of Psychology, University of Bath, Bath, UK

**Keywords:** Patient medicines helplines, National Health Service, Medicines information, Drug information, Hospital pharmacy, Hospital discharge, Medication errors, Qualitative, Framework analysis, RE-AIM

## Abstract

**Background:**

Patient medicines helpline services (PMHS) have been established at some National Health Service (NHS) Trusts in England, with the aim of providing medicines-related support to patients after they have been discharged. Addressing an important knowledge gap, this qualitative study sought to examine pharmacy professionals’ experiences and perceptions of their PMHS, including perceived benefits of the services, and areas for improvement.

**Methods:**

Invitations to participate were sent to all NHS Trusts within England that were known to provide a PMHS (*n* = 117). Semi-structured interviews were conducted via telephone with 34 pharmacy professionals who provide a PMHS (female = 76%, male = 24%; predominantly from Acute NHS Trusts, 76%). Interviews were audio-recorded and transcribed verbatim. The RE-AIM framework for evaluating interventions (RE-AIM: Reach, Effectiveness, Adoption, Implementation, Maintenance) informed the development of the interview schedule and the analysis of the data using framework analysis.

**Results:**

Two themes were generated from the analysis: *Resources,* and *Perceived benefits*. Findings illustrate how providing a PMHS with limited resources (e.g., no specific funding, understaffed) negatively impacts the implementation, maintenance and reach of PMHS, and the ability to evidence their effectiveness. Despite operating with limited resources, PMHS are considered to have many benefits for patients and healthcare organisations (e.g., providing a ‘safety net’ to patients during the transfer of care period, providing reassurance to patients, helping to optimise patients’ medicines, resolving medicines-related errors, reducing the burden upon other services, and providing the potential to improve hospital services based upon the content of enquiries). However, actually establishing the effectiveness and cost-effectiveness of PMHS is challenging due to perceived logistical difficulties of collecting data, and the difficulty measuring hard outcomes (e.g., prevention of readmissions).

**Conclusions:**

PMHS are typically perceived to be under-resourced, although they are considered by pharmacy professionals to have several benefits for service users and NHS Trusts. For those sites that provide a PMHS, we recommend using enquiry data to improve hospital services, and to share ideas for implementing and maintaining a PMHS within a resource-limited context. High-quality research is needed to evidence the effectiveness and cost-effectiveness of PMHS, which may help to secure adequate resources for this service in the future.

## Background

Patients often experience changes to their medicines regimen while they are in hospital, and healthcare policy in the United Kingdom (UK) requires that patients’ medicines are managed optimally after discharge from secondary care [1, 2]. However, a growing body of evidence highlights that a number of patients in the UK lack knowledge of their medications following hospital discharge [3], and report not receiving important information about their medications [4–7]. Additionally, findings suggest that up to 40% of patients who have been discharged from hospital may subsequently experience medicines-related problems or need support with their medicines [8–11].

Patient medicines helpline services (PMHS) have been set up by some National Health Service (NHS) Trusts^1^ in England, with the aim of providing support to recently discharged patients regarding changes to their medicines regimen as a result of their hospital care. The first PMHS was set up in 1992, and a survey study conducted in 2017 reported that 52% of NHS Trusts in England provided a PMHS [12]. A recent systematic review examined the evidence regarding the effectiveness of PMHS, concluding that PMHS are typically valued by service users (e.g., satisfaction ratings are excellent) and that the advice provided to service users is usually followed. Results of the review also identified that service users report several positive outcomes of consulting a PMHS which include resolution or avoidance of medicines-related problems, feeling reassured, and improved health [13]. This systematic review highlights that, to date, studies examining PMHS have mainly examined the views of service users, using quantitative methods.

PMHS are likely to be improved by seeking to understand the perceptions of not only service users but also service providers [14]. To date, the only studies to examine pharmacy professionals’ views of PMHS are survey studies examining how PMHS are provided [12, 15, 16]. For example, the most recent was an online survey conducted in 2017 that examined how PMHS are provided by NHS Trusts in England (*N* = 117) [12]. Findings showed that PMHS are under-used, under-promoted, and not sufficiently available to patients. Since the aim of the survey study was to provide a general overview as to how PMHS are provided, the authors did not seek to explore pharmacy professionals’ perceptions regarding the underuse of PMHS, nor why the implementation of PMHS are limited in several respects. This study also did not directly seek pharmacy professionals’ views regarding the effectiveness of PMHS. Instead, participants selected options from a list of perceived benefits that were compiled by three medicines information (MI) pharmacists [17], which may have biased the results.

Qualitative research can be important for understanding perceived reasons why healthcare services are effective or not, and how services can be improved [18, 19]. The idiographic approach to studying phenomena is unique to qualitative methods and enables greater importance to be placed on studying individuals' insights, understandings, and meanings of their lived experiences [20]. With the need to adopt a more in-depth and idiographic approach to exploring pharmacy professionals’ experiences and perceptions of providing a PMHS, qualitative methods are well suited to address this important issue.

### Aims

The aim of this novel study was to use qualitative methods to explore pharmacy professionals’ experiences and perceptions of providing a PMHS, in order to develop recommendations for service improvement for the benefit of users and providers of PMHS. In particular, this study addressed the following research question: *What are pharmacy professionals’ experiences and perceptions of providing an NHS patient medicines helpline service?*

## Method

### Study design

A qualitative interview design was chosen to explore pharmacy professionals’ experiences and perceptions of providing a PMHS. The authors adopted the epistemological position of pragmatism [21].

### Participants and recruitment

Participants were eligible for this study if they were either a Chief Pharmacist at an NHS Trust within England that operates a PMHS, or a pharmacy professional who operates a PMHS at their NHS Trust within England. Chief Pharmacists were invited to participate to provide a perspective as to how PMHS are beneficial within the wider organisation. Pharmacy professionals who operate a PMHS were invited to participate, since they see first-hand the benefits and potential limitations of this service. Eligible participants were required to be registered with the General Pharmaceutical Council (GPhC), which is the UK licensing body for pharmacy professionals.

Our estimated sample size was based upon that of published qualitative studies of healthcare professionals’ (HCPs) perceptions of healthcare services, and recommendations in literature [20]. We therefore aimed to conduct between 20 and 30 interviews. Upon reaching the thirtieth interview, we decided to invite the few remaining NHS Trusts that provide a PMHS in England who had not yet been contacted, to ensure that all relevant sites had the opportunity to participate (*n* = 117 Trusts, [12]). This resulted in a total of 34 participants.

Table 1 provides an overview of participant characteristics (see Additional file 1 for anonymised information regarding each participant). Most participants were female, and employed within an acute Trust.

**Table 1:** Participant characteristics

Characteristic		Participants (*n* = 34)
		n (%), or mean years (SD; range)
Gender	Male	8 (24%)
	Female	26 (76%)
Age		39 (9.50; 25 to 59)
Ethnicity	White or White British	27 (79%)
	Asian or Asian British	7 (21%)
Job title	Senior or Lead MI Pharmacist	16 (47%)
	Chief Pharmacist	4 (12%)
	MI Pharmacist	4 (12%)
	MI Manager	3 (9%)
	Pharmacist	3 (9%)
	Senior or Lead Pharmacist	2 (6%)
	Junior Pharmacist	1 (3%)
	Senior or Lead MI Technician	1 (3%)
Number of years employed as a pharmacy professional		16 (9.97; 3 to 35)
Number of years working on a PMHS		6 (3.71; 0.5 to 12)
NHS Trust type	Acute	26 (76%)
	Mental health	3 (9%)
	Integrated (two or more types)	2 (6%)
	Specialist	2 (6%)
	Community	1 (3%)

*Note*. Abbreviations: MI = medicines information; PMHS = patient medicines helpline service. NHS = National Health Service

### Data collection

Interviews were chosen as the data collection method in order to enable flexibility through the use of probes and unplanned questions. Telephone interviews were chosen to enable pharmacy professionals throughout England to be easily interviewed. Each participant was interviewed once.

An interview schedule was developed for the purpose of exploring participants experiences and perceptions of their PMHS, and was informed by the RE-AIM framework [22]. RE-AIM comprises five dimensions that are considered important for evaluating the impact of healthcare interventions: *Reach* (whether an intervention is reaching everyone who would benefit from it; perceived reasons for underuse); *Effectiveness* (the positive and negative consequences of an intervention); *Adoption* (whether an intervention is adopted by settings that could provide it; perceived reasons for or against adoption); *Implementation* (extent to which an intervention is delivered as intended); and *Maintenance* (extent to which an intervention becomes a stable, enduring part of the behavioural repertoire of an individual/organisation). We ensured that questions pertaining to each of the five RE-AIM dimensions were included in the schedule. For example, for *Adoption*, participants were asked the following: “Please could you describe why your patient medicines helpline service was developed?” (see Additional file 2 for the interview schedule). The topics of the interview schedule are presented in Table 2. The interview schedule was developed by the study authors in accordance with established conventions for semi-structured interviewing [23–25]. The interview schedule was not piloted. However, once drafted, the interview schedule was reviewed by three MI Pharmacists with expertise in operating a PMHS, with refinements made based upon their feedback.

**Table 2:** Main topics for interviews with pharmacy professionals regarding their patient medicines helpline service

Topics	
1. Perceived purpose of their patient medicines helpline service.	
2. Why the patient medicines helpline service was set up.	
3. Perceived qualities of a good patient medicines helpline service.	
4. How the quality of the patient medicines helpline service is ensured.	
5. Perceived benefits of operating a patient medicines helpline service.	
6. Perceived challenges of operating a patient medicines helpline service.	
7. Perceptions as to whether and in what ways the patient medicines helpline service meets service users’ needs.	
8. Perceptions as to whether and in what ways the patient medicines helpline service is cost-effective.	
9. Perceptions as to whether and in what ways any aspects of the patient medicines helpline service could be improved.	
10. Perceptions as to the usage of the patient medicines helpline service.	
11. Perceptions as to whether they feel they have all the resources needed to provide their patient medicines helpline service the way they want to.	
12. Perceptions as to procedures that are in place when an enquiry reveals that there has been a medicines-related error, and/or there is the potential to learn from an enquiry.	

During data collection, the interview schedule served as a flexible guide for interviews, enabling participants to discuss aspects of their PMHS that were important to them. All interviews were audio-recorded.

After their interview, the following background data were collected from each participant over the telephone: age, gender, ethnicity, job title, number of years employed as a pharmacy professional, and number of years’ experience of operating or providing a PMHS.

All data were collected by a trained interviewer (MW) between May and October 2018. Interview duration ranged from 16 to 53 min (mean = 30 min).

### Data analysis

All audio-recorded interviews were transcribed verbatim into separate Microsoft Word documents. Framework analysis (FA) was used to analyse the transcribed data. FA is a systematic, rigorous and transparent technique for organising, describing, and interpreting data, which has been used within health research [26–28]. FA was chosen instead of other thematic methods because the RE-AIM framework was to guide the analytic framework, and also because FA was developed to help manage relatively large qualitative datasets [27].

Analysis involved the following stages, as outlined by Ritchie and Spencer [29]: familiarisation with the data, coding, developing an analytical framework, indexing, charting, and interpretation. Being a flexible approach, FA can be used for deductive, inductive, or combined qualitative analysis [27]. A combined approach was used for the present study, whereby the five aspects of the RE-AIM framework were used as categories, and codes were developed from the data if they pertained to one of these five categories. However, inductive coding was also conducted as new concepts became apparent. All interview transcripts were uploaded into NVivo version 12 [30], which was used for the framework development and indexing stages. The only deviation to the FA stages was that Iterative Categorisation (IC) [31] was used in place of charting. The choice to use IC was made in order to increase transparency and rigour. IC leaves a clear audit trail, which provides a route back to the indexed data. With IC, each indexed code within the framework was exported from NVivo to a Microsoft Word document. Each document was then reviewed line-by-line, summarizing and organising the findings iteratively into points that represented commonalities and differences across participants. This ensured transparency as to which participants contributed to each point (see Neale [31] for further details). Final themes were generated after re-reading all of the IC documents. Study participants did not provide feedback on the findings, since evidence suggests that such checks may not improve study findings [32].

### Establishing quality in qualitative research

Yardley’s criteria for demonstrating the quality of qualitative research were met [33]. For *sensitivity to context*, previous literature was reviewed, and a theoretical framework (RE-AIM) was used to guide data collection and analysis. For *commitment and rigour*, FA and IC stages were followed, and a ‘paper trail’ approach was used. Credibility checks were conducted, where each stage of the analysis was checked by another member of the research team to verify that the identified codes and themes were appropriate. Additionally, the consolidated criteria for reporting qualitative research (COREQ) were followed [34]. For *coherence and transparency*, the study results are grounded in example quotations from the raw data. A reflective diary was used throughout the process of data collection and analysis, to record thoughts about each interview, contextual features that may have influenced interviews, and/or any ways that interviews could have been improved in order to enhance subsequent interviews. For *impact and importance*, the study findings were used to develop recommendations for improving the provision of PMHS.

## Results

Two themes were generated from the analysis: *Resources,* and *Perceived benefits*. *Resources* identifies that PMHS are often provided with limited resources. *Perceived benefits* identifies that PMHS are perceived to have several benefits, although evidencing the benefits is considered challenging.

### Resources

Participants described several resources that are considered useful for ensuring that they provide a high-quality PMHS. These included mechanisms for documenting enquiries, support of colleagues within the wider organisation (e.g., to promote the service), support of local and regional MI centres for conducting quality checks, and the use of standard operating procedures and training materials for new staff. Because PMHS function to resolve enquiries resulting from care received from secondary care services, participants expressed the need for local, Trust-based resources to achieve this (e.g., discharge summaries, blood results, drug charts, inpatient notes, access to HCPs involved in their care). Some participants described how their service is also available for patients of neighbouring Trusts, and that lacking local resources to answer enquiries may result in delayed help for these patients.

“*We offer the service to other places now… We don’t have access to their patient records, which is not ideal for operating a patient helpline. But we have a system in place where, if we need to look at the records, we have a contact to ring... And they will have a look at, say, the patient’s discharge letter and perhaps fax that over to us or email it to us*.” (P1, Lead MI Pharmacist, Acute Trust).

Participants perceived one of the biggest challenges for adopting, implementing and maintaining a PMHS to be inadequate funding and resources. Many participants described the lack of specific funding and resources for their service.

“*We initially formed and set up the service and took it live with no additional cost to the pharmacy department. It was an additional role added in to the portfolio of our medicines information department*.” (P4, Chief Pharmacist, Acute Trust).

Some participants described the difficulty in obtaining funding due to the low number of enquiries received to the service. This suggests a difficult situation where the numbers of enquiries are too small to justify additional funding/resources, yet without additional funding/resources, the number of callers is unlikely to increase.

“*I think it would be difficult with the number of enquiries we get to say that there is, you know, a pressure. Or to support a business case for getting additional staff*.” (P13, Senior MI Pharmacist, Acute Trust).

Although some participants considered their PMHS to be inexpensive to run, other participants described that funding is needed, and that not having funding has a negative impact upon the service.

“*I think it’s probably as good as it could be, given the amount they’re investing in it, to be honest. So, what they’re prepared to put in, they’ll get out.*” (P16, Pharmacist, Mental Health Trust).

A consequence of limited funding/resources is that the availability of PMHS may be inadequate. Some providers acknowledged that they do not currently meet national standards for satisfactory PMHS availability (i.e., available for at least 4 hours a day, 5 days a week), but that some provision is better than none. At some sites, pharmacy professionals described how, even when their helpline should be manned, they may not always be available to answer enquiries due to other commitments and staffing issues***.***

“*You’ve got discharges to do, new patients to see, high risk patients, and you’ve got those that have been discharged that have phoned up with queries. So, it’s kind of going into more of a clinical pot to be prioritised and answered at some point during the day*.” (P20, Lead Pharmacist, Specialist Trust).

At some sites, the enquiries are received only by voicemail, which means that a caller will never directly speak to a pharmacy professional when they contact the service for help. Providing a service with limited access and availability, such as a voicemail service, was acknowledged as having potential negative consequences. For example, callers may feel frustrated and seek advice elsewhere, or not seek help at all. Limited access and availability may therefore also have an impact upon the use of the service.

“*I think being able to have someone answering the phone more consistently throughout the day [would improve its use]… I think if there’s no-one there to answer the phone, then people don’t leave a message. So, I think we are probably missing people because of that*.” (P13, Senior MI Pharmacist; Acute Trust).

Since the majority of enquiries to PMHS were considered to be relatively straightforward, some participants described that a triaging system would be cheaper and more efficient, whereby a pharmacy technician receives and answers enquiries, and forwards more complex ones to a pharmacist. Some sites had already implemented such a model.

Lack of resources was also discussed in relation to the perceived under-promotion of PMHS. Most participants commented that their service is not promoted enough, and in some cases, not promoted at all. Reasons included not having the time to monitor the advertising, and fear of over-promoting the service and not being able to cope with the demand.

“*It was decided that we would just promote through cards [given out with prescriptions]. We didn’t want to sort of over-promote the service and then have me drowning in enquiries. Because I’m on my own*.” (P7, MI Pharmacist, Acute Trust).

This suggests that, due to lack of time/resources, some PMHS providers are purposefully restricting the use of their service. However, this may mean that some patients will not learn of the existence of the service, which may result in them being inconvenienced or suffering as a consequence of improper use of their medicines. Participants commented upon the link between the lack of promotion of their service, and its perceived underuse.

“*I think it could be more widely used, if we found ways of promoting it better. So, we need to make sure that the number is on every piece of paper that comes out of any department. And then I think we would have more uptake*.” (P15, MI Manager, Acute Trust).

Some participants described the benefit of particular promotional methods. For example, promoting the service via the discharge summary is not only free advertising, but it also ensures that the service is promoted to every discharged patient, which increases the number of enquiries.

Despite often providing a PMHS with limited resources, most participants commented that answering enquiries to their PMHS and helping patients provides them with job satisfaction. This could be a motivation for continuing to run a PMHS, despite limited resources.

*“We love providing the service. We really do…When you’ve resolved something for a patient, even if it’s very simple for us, obviously it’s concerned them enough to give you a ring. When you’ve resolved an issue, it actually kind of leaves you with quite a nice fuzzy feeling.”* (P26, Lead MI Pharmacist, Acute Trust).

### Perceived benefits

Participants described several perceived benefits of providing a PMHS. Primarily, PMHS were perceived to be beneficial for providing patients with personalised, expert support during the transfer of care period. PMHS were considered necessary in order to address often inadequate discharge processes in which discharge counselling is limited. Discharges were perceived to be inadequate for several reasons, such as the difficulty of patients/carers retaining a large amount of information provided on discharge, HCPs having insufficient time to explain everything, and discharges occurring out-of-hours when HCPs are often unavailable.

“*It’s a safety net. The pharmacists are quite time-pressured on the wards but unfortunately counselling of patients on discharge probably doesn’t happen as much as we would like. So, the advice line helps sort of mop up any missed important counselling points really, for those proactive patients that call us*.” (P2, Lead MI Pharmacist, Acute Trust).

The above quotation also highlights that PMHS may not reach all patients who require support, since the onus is upon the patient or carer making contact with the service. It is likely that there is an unknown number of patients who require support with their medicines after discharge but who are *not* proactive and do not call the hospital’s PMHS.

Perceived benefits upon patients and carers also included improving patients’ adherence and knowledge of their medicines, helping patients take medicines safely and therefore potentially avoiding harm, and improving patients’ experiences of care. No participants described any perceived adverse effects of providing a PMHS.

“*[A benefit of the helpline is] them continuing to take the right medicines at the right times in the right way, meaning that they have the best outcomes and have the most, you know, optimal use of their medicines*.” (P21, Lead MI Pharmacist, Acute Trust).

Offering a helpline also enabled pharmacists to provide reassurance to patients who have queries about their medication. This was considered to be an important function, and may be pivotal in encouraging patients to use medication appropriately.

“*Sometimes, people just want some reassurance. We know that patients don’t often take their medications as they’re prescribed. And actually, often that can be due to kind of misconceptions that they have about their medicines. Or concerns. Being able to speak to someone in a bit more of a calm environment than a ward post-discharge, can be all patients need sometimes to consider continuing to take their medicines*.” (P33, MI Manager, Acute Trust).

Extending the benefits of PMHS beyond the individual level, participants described how PMHS successfully sought to positively promote pharmacy services and the Trust at a wider level (e.g., showing continued responsibility after discharge).

“*I think it [PMHS] sheds a positive light on to the Trust. It shows that the Trust cares about their patients. And you know, their level of responsibility doesn’t end when the patients are physically discharged*.” (P12, Pharmacist, Mental Health Trust).

Participants also described the PMHS as being a mechanism for catching and/or resolving medicines-related errors, which can provide a learning and improvement opportunity for the Trust.

*“Sometimes queries do bring out that there has been an error. So sometimes patients have got home and they haven’t got something that they should’ve had, or they’ve got somebody else’s something that they shouldn’t have. So, it’s making sure that those errors are fed back to the appropriate people and filling in the appropriate incident reporting forms, as well.”* (P21, Lead MI Pharmacist, Acute Trust).

Participants also described how use of PMHS could benefit the trust more widely though acting as a mechanism for fixing medication-related issues before they result in formal complaints to the Trust.

*“You would hope you’d have less formal complaints. Because it’s the first place that people come to, we can hopefully resolve any queries that they’ve got before, you know, they escalate in to something else whereby they then need to make a more formal complaint.”* (P14, Chief Pharmacist, Acute Trust).

Other perceived benefits for the Trust included preventing hospital readmissions, reducing the burden upon other services, and the potential to learn from enquiries in order to improve services (e.g., examining trends in the types of enquiries received to improving discharge counselling and/or patient information leaflets).

**“***We can collect [enquiries], and where we get a trend, what we’ll do is we’ll go to the department and say “We’ve had a fair number of enquiries about this. You might want to have a rethink about your system*.”” (Participant 29, MI Pharmacist, Acute NHS Trust).

Although several perceived benefits were described, many participants reported being unsure regarding the *actual* effectiveness of their PMHS, since the effectiveness of their service had not been evaluated. Measuring the effectiveness of PMHS was considered to be problematic for numerous reasons including difficulties with collecting data from PMHS users (e.g., lack of time and resources, difficulty obtaining consent, and previous response rates being low).

“*We are supposed to send out questionnaires to patients who have used the service to find out about their experiences… It’s very very rare that any of them respond*.” (P34, Senior MI Pharmacist, Acute Trust).

The following quotation also highlights how, for some Trusts, the underuse of PMHS can hinder evidencing the effectiveness of the service, since it may be difficult to collect meaningful data from a small sample.

“*We’ve definitely not done any sort of studies looking at [the effectiveness of the service]. And our numbers are probably too small for it to really be significant details*.” (P9, Chief Pharmacist, Specialist Trust).

In addition to the challenges around the logistics of collecting data, participants also described the difficulty of measuring effectiveness and cost-effectiveness in order to evidence that providing their PMHS is worthwhile (e.g., the difficulty evidencing what would have happened had the service not existed, the difficulty evidencing that the service reduces readmissions and/or the burden upon other healthcare services, and the difficulty measuring actual outcomes for patients instead of their perceived outcomes).

“*To actually benchmark and measure better outcomes is incredibly difficult. How do you actually show to the Trust that Fred Bloggs… how do you actually demonstrate that his outcomes were better because he had that intervention? … It’s very difficult for the Trust to know that that service is good.*” (P18, Chief Pharmacist, Integrated Trust).

Despite this, participants commented on the importance of using enquiry data to show the value of their PMHS. For example, performance statistics (e.g., number of enquiries received per month, types of enquiries) can be produced for the Trusts’ pharmacy dashboard, senior managers, and the hospital executive team.

## Discussion

This study explored the experiences and perceptions of providing a PMHS in a sample comprising 34 pharmacy professionals. Two themes were generated: *Resources*, and *Perceived benefits*. The findings illustrate how providing a PMHS with limited resources (e.g., specific funding, adequate staffing) impacts upon their implementation, maintenance, reach, and the ability to evidence their effectiveness. Despite this, PMHS are considered to have a number of benefits for patients and healthcare organisations (e.g., providing a ‘safety net’ to patients during the transfer of care period, providing reassurance, helping to optimise patients’ medicines, preventing readmissions, and reducing the burden upon other healthcare services). However, actually establishing the effectiveness and cost-effectiveness of PMHS is challenging due to perceived logistical difficulties of collecting data, and the difficulty measuring hard outcomes.

Our finding that PMHS are often provided with limited resources corresponds with a recent survey study showing that the main reason why 48% of NHS Trusts in England in 2017 did not provide a PMHS was also due to lack of staffing/resources [12]. Our findings also suggest that a particular challenge for proving a PMHS was having pharmacy professionals being available to answer calls during the working day. This accords with findings of the same survey study, showing that, although 86% of Trusts that provide a PMHS reported it as being accessible for at least 4 hours per day, only 57% of Trusts reported that their PMHS was accessible for eight or more hours per day. Additionally, 29% of Trusts reported that contact with a pharmacy professional is not always available during advertised hours.

The lack of access and availability of PMHS may mean that patients choose to seek advice elsewhere, increasing the burden upon primary care. A recently conducted systematic review found that, if a PMHS was not available, patients and carers would most likely seek the advice of their GP instead [13]. This is topical, given that the average waiting time from booking a standard appointment to seeing a GP in England in 2016 and 2017 was approximately 2 weeks [35]. A delay to receive appropriate medicines-related support may have implications for patients regarding the optimisation of their medicines and their ability to be adherent, which may impact their wellbeing. Patients and carers also have other options, such as visiting a community pharmacist or contacting NHS 111 (a general health information service in England). However, Badiani et al. found that, out of 200 enquiries received to their PMHS, 74.5% required access to hospital-based resources (e.g., patient records, and healthcare providers) [36]. Thus, community pharmacists and NHS 111 are currently unlikely to be able to answer the majority of enquiries.

Our findings suggest that, from the perspectives of pharmacy professionals, PMHS have a number of benefits for patients and the healthcare organisation. These perceived benefits are consistent with the outcomes reported in a recently conducted systematic review examining the effectiveness of PMHS (e.g., patients feeling reassured, patients’ improved use of medicines) [13]. However, the outcomes in this systematic review are also based upon perceptions, since the identified studies primarily involved surveying service users, or having clinicians rate enquiries and answers in order to hypothesise the impact upon patients. Therefore, to date, no studies have examined hard outcomes from using a PMHS (e.g., readmissions). This accords with our finding that pharmacy professionals view measuring hard outcomes from PMHS as challenging.

Participants’ description of their PMHS as a ‘safety net’ for supporting patients who had experienced a medicines-related error is consistent with findings regarding error rates following hospital discharge. Approximately 40% of patients may experience medicines-related errors after discharge from hospital [37, 38]. Learning from medicines-related errors in order to implement methods for their reduction is a current NHS and worldwide healthcare priority [39]. In relation to PMHS, a recent systematic review examined the types of enquiries received to PMHS and found that, on average, 27% of calls are regarding medicines-related errors [40]. Therefore, a PMHS may provide one avenue for reducing medicines-related errors, if the information from such enquiries is developed into recommendations and implemented to improve practice.

Our findings suggest that a consequence of reducing medicines-related errors and improving medicines-related counselling at hospital discharge may be that fewer patients need support with their medicines following hospital discharge. This may result in the number of enquiries to PMHS being reduced. Reducing the need for patients to contact a PMHS after their discharge may help to address one of the key issues of providing a PMHS that was found in our study – that resources are often insufficient. Additionally, providing better medicines-related support to patients earlier in their care pathways could mean that more patients avoid medicines-related issues after discharge, and not just the proactive patients and carers who choose to seek support.

### Recommendations for practice

Our findings suggest that recommendations to improve the impact of PMHS must be achievable within a resource-limited context. Therefore, we recommend the following:

PMHS providers could examine the types of calls they receive, and where possible, learn from them and improve practice. Over time, certain enquiries (e.g., those regarding errors, and those received as a consequence of inadequate discharge counselling) may be reduced, which may also reduce the burden upon PMHS providers.

To reduce the cost of manning a PMHS, rather than having a pharmacist answer enquiries, a triaging system would be cheaper and more efficient whereby a pharmacy technician answers enquiries, and forwards more complex ones to a pharmacist.

In order to reduce the cost of promotion, and increase the reach of PMHS to all discharged hospital patients who may require support with their medicines, helpline providers could promote their service for free by ensuring that their helpline number is included within discharge summaries that patients receive.

Relatedly, we recommend that providers of PMHS share their ideas for implementing and maintaining a PMHS within a resource-limited context. This could be co-ordinated by regional MI centres, and published via the UK medicines information network [41]. We also recommend that outputs from all improvement projects are made available by PMHS providers to their senior managers and their hospitals’ executive team, in order to show the value and efficiency of providing a PMHS.

### Recommendations for future research

High-quality, multi-site research is needed to examine whether the perceived benefits of PMHS described in this study can be evidenced. Such evidence may result in more NHS Trusts establishing their own PMHS. This is important, since a survey study conducted in 2017 found that only 52% of Trusts in England provided a PMHS [12]. However, evaluation of PMHS services was a critical issue in the present study, with participants describing difficulties measuring the effectiveness and cost-effectiveness of PMHS services. In order to share expertise and resources, and increase the generalisability of findings, we recommend that sites collaborate with one another, for example, within a region. Such studies could be coordinated by regional MI centres.

The views and experiences of both service users’ and service providers’ are considered important for improving the quality of healthcare services [14]. It would therefore also be advantageous for future research to explore patients’ and carers’ experiences of using a PMHS, also using qualitative methods. Such an idiographic approach would enable service users themselves to provide a detailed consideration of how and why PMHS may be beneficial, and ways that they may be improved, in order to develop further recommendations for their improvement.

### Strengths and limitations

This is the first study to take an idiographic approach to exploring pharmacy professionals’ perceptions of PMHS, thereby providing rich and contextualised accounts of PMHS provision that have resulted in recommendations for service improvement and future research endeavours. Additionally, we used RE-AIM, an established evaluation framework, to achieve this [22]. Thus, all five aspects of the impact of interventions, as conceptualised by the developers of RE-AIM, have been incorporated into our data collection and analysis processes. Consideration was also made throughout the study processes to enhance the validity and trustworthiness of our findings.

A limitation of this study is that although we invited pharmacy professionals from all known NHS Trusts that provided a PMHS, pharmacy professionals were still required to opt in to participate. This may have resulted in bias, since the perspectives of the pharmacy professionals who decided not to participate are not represented. Additionally, the sample predominantly comprised pharmacy professionals from acute NHS Trusts, and therefore could have be improved by the addition of pharmacy professionals from other Trust types (e.g., mental health, specialist, and community Trusts).

## Conclusion

This qualitative study highlights several potential benefits of PMHS, for both patients and healthcare organisations. However, actually evidencing the benefits of PMHS is perceived to be challenging, such as the difficulty measuring what would have happened had the service not existed, and not having the resources to evidence their effectiveness. Lack of resources (e.g., no specific funding, staffing) was also perceived to impact the implementation, maintenance, and reach of PMHS. We recommend that helpline providers share best practice for providing a PMHS within a resource-limited context. High-quality research is needed to evidence the effectiveness and cost-effectiveness of PMHS, which may help to secure adequate resources for this service in the future.

## Supplementary information

**Additional file 1.** Participant characteristics. Table providing a more detailed account of participant characteristics.

**Additional file 2.** Interview schedule. Data collection interview schedule.

## Data Availability

Anonymised transcripts and datasets are available from the University of Bath Research Data Archive to bona fide researchers [43].

## Abbreviations

PMHS: Patient medicines helpline service
UK: United Kingdom
NHS: National Health Service
RE-AIM: Reach, efficacy/effectiveness, adoption, implementation, and maintenance
MI: Medicines information
GP: General practitioner
A&E: Accident and emergency department
