# Supplementary material for: Pharmacy professionals’ experiences and perceptions of providing NHS patient medicines helpline services: a qualitative study
Source: BMC Health Serv Res. 2020 Apr 29;20:364. doi: 10.1186/s12913-020-05182-w (PMC7189450; doi:10.1186/s12913-020-05182-w)
Supplement: Supplementary file 1 — Additional file 1. Participant characteristics. Table providing a more detailed account of participant characteristics. [file 12913_2020_5182_MOESM1_ESM.docx]

| *Supplementary Table 1. Participant characteristics (detailed)* | | | | | |
| --- | --- | --- | --- | --- | --- |
| Participant number | Gender | Job title | Years employed as a pharmacy professional | Years operating a PMHS | NHS Trust type |
| P1 | Female | Lead MI Pharmacist | 35 | 8 | Acute |
| P2 | Female | Lead MI Pharmacist | 6 | 6 | Acute |
| P3 | Female | MI Manager | 21 | 6 | Acute |
| P4 | Male | Chief Pharmacist | 30 | 6 | Acute |
| P5 | Male | Lead MI Pharmacist | 10 | 4 | Acute |
| P6 | Female | Lead MI Pharmacist | 14 | 7 | Acute |
| P7 | Female | MI Pharmacist | 6 | 2 | Acute |
| P8 | Male | Pharmacist | 3 | 6 months | Acute |
| P9 | Female | Chief Pharmacist | 18 | 9 | Specialist |
| P10 | Female | Lead MI Pharmacist | 7 | 6 | Acute |
| P11 | Female | MI Pharmacist | 4 | 2 | Acute |
| P12 | Female | Pharmacist | 23 | 5 | Mental health |
| P13 | Female | Senior MI Pharmacist | 19 | 10 | Acute |
| P14 | Female | Chief Pharmacist | 33 | 5 | Acute |
| P15 | Female | MI Manager | 25 | 10 | Acute |
| P16 | Male | Pharmacist | 3 | 9 months | Mental health |
| P17 | Female | Lead MI Technician | 26 | 3.5 | Integrated |
| P18 | Female | Chief Pharmacist | 37 | 4 | Integrated |
| P19 | Female | MI Pharmacist | 3 | 1 | Acute |
| P20 | Female | Lead Pharmacist | 20 | 12 | Specialist |
| P21 | Female | Lead MI Pharmacist | 15 | 1.5 | Acute |
| P22 | Male | Senior MI Pharmacist | 19 | 5 | Acute |
| P23 | Female | Lead MI Pharmacist | 8 | 5 | Acute |
| P24 | Female | Lead MI Pharmacist | 22 | 7 | Acute |
| P25 | Female | Lead MI Pharmacist | 7 | 5.5 | Acute |
| P26 | Female | Lead MI Pharmacist | 13 | 5 | Acute |
| P27 | Male | Lead MI Pharmacist | 15 | 2 | Community |
| P28 | Female | Lead MI Pharmacist | 12 | 3 | Acute |
| P29 | Female | MI Pharmacist | 21 | 19 | Acute |
| P30 | Female | Senior Pharmacist | 28 | 5 | Mental health |
| P31 | Male | Junior Pharmacist | 3 | 2.5 | Acute |
| P32 | Female | Lead MI Pharmacist | 17 | 7 | Acute |
| P33 | Male | MI Manager | 7 | 2 | Acute |
| P34 | Female | Senior MI Pharmacist | 6 | 3 | Acute |
| *Note*. Abbreviations: PMHS = patient medicines helpline service; MI = medicines information; NHS = National Health Service. | | | | | |
