# Supplementary material for: Pharmacy professionals’ experiences and perceptions of providing NHS patient medicines helpline services: a qualitative study
Source: BMC Health Serv Res. 2020 Apr 29;20:364. doi: 10.1186/s12913-020-05182-w (PMC7189450; doi:10.1186/s12913-020-05182-w)
Supplement: Supplementary file 2 — Additional file 2. Interview schedule. Data collection interview schedule. [file 12913_2020_5182_MOESM2_ESM.docx]

| **Q1** | **To begin, could you please tell me about your role within your NHS Trust’s Pharmacy Services?** |
| --- | --- |
| **Q2** | **Please could you tell me about your role in relation to the patient medicines helpline service?** |
|  | Probe – [For pharmacists, not chief pharmacists] Please could you tell me your responsibilities in terms of the patient medicines helpline?  Probe – [For both pharmacists and chief pharmacists] Did you help to set up the patient medicines helpline? If so, please could you describe what your involvement was in setting the service up? |
| **Q3** | **Please could you describe why your patient medicines helpline service was developed?** |
|  | Probe – Research studies? Guidelines? (If so, which?) Awareness of national standards?  Probe – What do you consider to be the purpose of the patient medicines helpline service? (Has its purpose changed over time? If so, in what ways? And Why?) |
| **Q4** | **Please could you tell me your thoughts about your patient medicines helpline service?** |
|  | For example, meeting its aims.  Probe - Is there anything you’d like to say about the cost of running the service? Probe - Is there anything you’d like to say about how the service may have developed since it first began? |
| **Q5** | **Please could you tell me what you consider to be the positive aspects of operating a patient medicines helpline service?** |
|  | For example… Probe – Aspects that are positive for service users.  Probe – Aspects that are positive for you/the MI team (e.g., learning from adverse patient experiences). Probe – Aspects that are positive for the hospital/NHS Trust (e.g., learning from adverse patient experiences; service improvement initiatives as a result of operating a PMH). Probe – Are there any other positive aspects of operating your patient medicines helpline which you haven’t mentioned so far? |
| **Q6** | **Please could you tell me about any challenges of operating a patient medicines helpline service?** |
|  | For example… Probe – Aspects that are negative/unhelpful/risks/safety issues for service users. Probe – Aspects that are negative/unhelpful for you/the MI team Probe – Aspects that are negative/unhelpful for the hospital/NHS Trust. Probe – Are there any other challenges of operating your patient medicines helpline service which you haven’t mentioned so far? |
| **Q7** | **Please can you tell me your thoughts regarding whether the patient medicines helpline service meets the needs of patients and carers?** |
|  | For example, their medicines information needs; their support needs. Probe – Please could you explain why?/why not? Probe – Is there anything you’d like to say about the uptake of the service? (If they mention lack of use: Probe - Do you have any suggestions as to why this might be? Probe – Lack of promotion; lack of need; use of other services (if so, which?)). Probe – Is there anything you’d like to say about the types of patients/people who use the service?  Probe – Are there any types of patients/people who you think could benefit from the service but who don’t typically use it? (If so, who? And why?) |
| *(If not already known from previous answers)* | |
| **Q8** | **Can you tell me your thoughts regarding the cost-effectiveness of the patient medicines helpline?** |
|  | Probe – *(If not already known)* Please could you say how your helpline service is funded? |
| **Q9** | **Please could you tell me about any aspects of your patient medicines helpline service that you think could be improved?** |
|  | Probe - If so, why? / In what way?  *For example….* Probe – Service user access. (Alternative methods? Online chat? Email? Skype / facetime?) Probe – Helpline availability. Probe – Helpline promotion. Probe – Procedures you use (e.g., documenting the calls; use of a SOP). Probe –IT systems and technology you use.  Probe – Service user involvement (e.g., including feedback/satisfaction surveys). Probe – Mechanisms to feed back to the Trust any issues/errors which become apparent during the operation of the service.  Probe – (If not already mentioned) Are you aware of the national standards which are available for operating a patient medicines helpline service? Have you used them to develop your service? If so, in what ways? |
| **Q10** | **Research suggests that sometimes people contact a patient medicines helpline service if there is an error with their medicines.** **Please could you describe any process that occurs if a helpline call reveals that an error has been made with a patient’s medicines?** |
|  | Probe – Learning from errors / Service improvement initiatives. |
| **Q11** | **In what ways do you think the helpline service could be used to improve practice, within Pharmacy Services and the wider organisation?** |
|  | Probe – For example, if data is routinely collected about errors, using that information to improve practice. Probe – [If not yet known] Based upon the examples you’ve given, are these means of improvement used at your hospital to improve practice? |
| **Q12** | **What qualities do you perceive to be important in order to provide a successful patient medicines helpline service?** |
|  | Probe – (if not obvious) Why are these things important? Probe – What staff skills are important? (Training? Standard operating procedures?) |
| **Q13** | **How do you see patient medicines helplines at NHS Trusts developing in the future?** |
|  | Probe – Developing the service at your NHS Trust, specifically (e.g., additional ways of accessing the service; availability of the service; promotion; procedures; IT/technology to provide the service; service user involvement). Probe – Developments within the UK generally (e.g., move towards regional/shared services - Carter Report / 5 year forward review / sustainability and transformation plans). Probe – Any perceived financial/funding issues in the future. |
| **Q14** | **In what ways is local knowledge needed in order to deal with helpline enquiries? By ‘local knowledge’, I mean knowledge that is available at your hospital or NHS Trust only (e.g., patient records, local policies and procedures, advice from clinicians who cared for the patient).** |
|  | Probe – patient records; access to relevant clinical staff; local policies and procedures. |
| **Q15** | **What do you think the impact would be if patient medicines helpline services became regional or national in the future, instead of local to hospitals?** |
|  | Probe – Impact of this upon the service / quality of the service / the type of information that could be provided? |
| **Q16** | **Those are all of the questions that I have about patient medicines helpline services. Although, is there anything else which you would like to say about patient medicines helpline services, which you feel would be important to share at this point?** |
